# Benzyl-2-Acetamido-2-Deoxy-α-d-Galactopyranoside Increases Human Immunodeficiency Virus Replication and Viral Outgrowth Efficacy *In Vitro*

**DOI:** 10.3389/fimmu.2017.02010

**Published:** 2018-01-26

**Authors:** Alex Olvera, Javier P. Martinez, Maria Casadellà, Anuska Llano, Míriam Rosás, Beatriz Mothe, Marta Ruiz-Riol, Gemma Arsequell, Gregorio Valencia, Marc Noguera-Julian, Roger Paredes, Andreas Meyerhans, Christian Brander

**Affiliations:** ^1^IrsiCaixa – AIDS Research Institute, Badalona, Barcelona, Spain; ^2^Universitat de Vic-Universitat Central de Catalunya (UVic-UCC), Vic, Barcelona, Spain; ^3^Infection Biology Group, Department of Experimental and Health Sciences, University Pompeu Fabra, Barcelona, Spain; ^4^Unitat VIH, Hospital Universitari Germans Trias i Pujol, Badalona, Barcelona, Spain; ^5^Institut de Química Avançada de Catalunya (IQAC-CSIC), Barcelona, Spain; ^6^Universitat Autonoma de Barcelona, Cerdanyola del Vallès, Barcelona, Spain; ^7^Institució Catalana de Recerca i Estudis Avançats (ICREA), Barcelona, Spain

**Keywords:** human immunodeficiency virus-1, benzyl-2-acetamido-2-deoxy-α-d-galactopyranoside, O-glycosylation, viral outgrowth, replication, infectivity

## Abstract

Glycosylation of host and viral proteins is an important posttranslational modification needed to ensure correct function of glycoproteins. For this reason, we asked whether inhibition of O-glycosylation during human immunodeficiency virus (HIV) *in vitro* replication could affect HIV infectivity and replication rates. We used benzyl-2-acetamido-2-deoxy-α-d-galactopyranoside (BAGN), a compound that has been widely used to inhibit O*-*glycosylation in several cell lines. Pretreatment and culture of PHA-blast target cells with BAGN increased the percentage of HIV-infected cells (7.6-fold, *p* = 0.0115), the per-cell amount of HIV p24 protein (1.3-fold, *p* = 0.2475), and the viral particles in culture supernatants (7.1-fold, *p* = 0.0029) compared to BAGN-free cultures. Initiating infection with virus previously grown in the presence of BAGN further increased percentage of infected cells (30-fold, *p* < 0.0001), intracellular p24 (1.5-fold, *p* = 0.0433), and secreted viral particles (74-fold, *p* < 0.0001). BAGN-treated target cells showed less CD25 and CCR5 expression, but increased HLA-DR surface expression, which positively correlated with the number of infected cells. Importantly, BAGN improved viral outgrowth kinetics in 66% of the samples tested, including samples from HIV controllers and subjects in whom no virus could be expanded in the absence of BAGN. Sequencing of the isolated virus indicated no skewing of viral quasi-species populations when compared to BAGN-free culture conditions. BAGN also increased virus production in the ACH2 latency model when used together with latency-reversing agents. Taken together, our results identify BAGN treatment as a simple strategy to improve viral outgrowth *in vitro* and may provide novel insights into host restriction mechanisms and O-glycosylation-related therapeutic targets for HIV control strategies.

## Introduction

Posttranslational modifications play a key role in folding, stabilization, trafficking, and function of eukaryotic proteins (1). Such modifications are also needed to ensure the correct function of viral proteins within infected cells or viral particles. Glycosylation is one of the most abundant and heterogeneous posttranslational modifications (2, 3) and is divided into two main types: (i) N*-*glycosylation, were N*-*glycans are bound to the –NH_2_ moiety of an asparagine (N) and (ii) O*-*glycosylation, where the –OH moiety of serines (S) or threonines (T) is modified by the addition of O*-*glycans. The role of N*-*glycosylation in human immunodeficiency virus (HIV) infection has been studied in detail, since it has major implications for the recognition of the viral envelope (Env) protein by broadly neutralizing antibodies (4–6). In comparison, little is known about the impact of O*-*glycosylation of host and viral proteins on HIV infection and replication, despite its potentially important role for proper protein function and viral life cycle (7).

During protein O*-*glycosylation, O*-*glycans are covalently α-linked *via* an *N-*acetylgalactosamine (GalNAc) moiety to serine or threonine residues using an O*-*glycosidic bond (2, 3). The *N-*acetylgalactosamine is usually extended by the addition of more glycans to create a heavily O-glycosylated glycoprotein named mucin, which are branched and markedly heterogeneous. Mucins are usually secreted or transmembrane glycoproteins, ubiquitous in mucous secretions and epithelial surfaces of the human body, including the gastrointestinal, genitourinary, and respiratory tracts. There, one of their functions is to shield epithelial surfaces against physical and chemical damage and protect against infection by pathogens (2).

In the context of a HIV infection, a number of studies have addressed the effect of secreted mucin glycoproteins on virus infectivity (8). Habte et al. showed that human saliva inhibits HIV infection (8, 9) and that salivary (10), breast milk (11), and cervical or pregnancy plug mucins (12) all inhibit HIV-1 infectivity in an *in vitro* inhibition assay (8, 9). Additional reports also indicate that members of the *T cell immunoglobulin and mucin* (TIM) protein family have an antiviral effect (13). However, these studies focused on the antiviral effects of the glycoprotein as a whole, not of the glycan moiety *per se*, and there is little information about the effect of O*-*glycosylation on HIV’s main cellular target (CD4+ T cell) or on viral protein function.

In contrast to the numerous predicted and experimentally defined N*-*linked glycosylation sites in the viral surface glycoprotein Env, there are only two threonine residues, at positions 499 and 606 that might undergo O-glycosylation (14–17). However, whether they are both glycosylated is questionable as it has been reported that Env proteins in HIV-1 and SIV virions lacked any detectable O-glycosylation of the C-terminal threonine (18). On the other hand, viral particles also carry, in their cell-derived membrane, a myriad of host proteins (19), such as HLA-DR, HSP90, RAC2, RHOA, or TIM-1 (13), many of which can be O*-*glycosylated (www.uniprot.org/uniprot).

Taken this into consideration, we hypothesized that interference with O*-*glycosylation processes could affect *in vitro* viral infectivity and replication. To test the effects of altered O-glycosylation on HIV replication, we used one of the best-studied inhibitors of mucin biosynthesis, benzyl-2-acetamido-2-deoxy-α-d-galactopyranoside (BAGN), which has been shown to inhibit O*-*glycosylation in a variety of cell lines (20–24). BAGN acts as a GalNAc-α-1-O-serine/theonine mimic and thus as a competitive inhibitor of O*-*glycan chain extension by blocking the β1,3-galactosyltransferase involved in O*-*glycosylation elongation (23, 25). As a result, BAGN is thought to inhibit mucin synthesis, although it does not prevent the initial synthesis of the core glycans (24, 26, 27). Additionally, this compound has been shown to have some other effects, including the inhibition of sialylation and disrupting the secretion of glycoproteins in HT-29 cells (22, 28). We used BAGN in different combinations with viral stocks and PHA-blast target cells to assess the impact that O*-*glycosylation inhibition may have on HIV infectivity and replication *in vitro*.

## Materials and Methods

### Peripheral Blood Mononuclear Cells (PBMC) Isolation and PHA Activation

Peripheral blood mononuclear cells were isolated from whole blood samples by using a Lymphoprep™ (Stemcell) density gradient and were frozen until use. To activate PBMC prior to infection with HIV, cells were thawed and cultured for 3 days in phytohemagglutinin (PHA, 5 µg/ml) containing R20/50 medium. R20/50 medium consisted of Roswell Park Memorial Institute (RPMI) 1640 (Gibco) supplemented with 2 mM l-glutamine (Gibco), 100 U/ml penicillin, 100 µg/ml streptomycin (Gibco), 20% of fetal calf sera (Invitrogen), and 50 U/ml of interleukin 2 (IL-2, Roche). The 3-days PHA-blasts were used as target cells in infection assays either unmodified or pre-incubated overnight with BAGN. PBMC were obtained from HIV sero-negative and from HIV-infected, treatment naïve, donors. Before obtaining any samples, donors signed informed consent forms approved by the Ethics Committee of the Hospital Universitari Germans Trias i Pujol (Badalona, Spain).

### Infection of PHA-Activated PBMC in the Presence of BAGN

To test the effect of BAGN on HIV *in vitro* infection, PHA-blasts from HIV sero-negative donors were generated. One-half of the target cells were pretreated by adding 2 mM BAGN (Sigma) to the PHA-blast at day 2 after PHA stimulation, i.e., BAGN was added overnight from day 2 to 3. At day 3, both BAGN pretreated and non-pretreated PHA-blast were pelleted by centrifugation at 400 *g* for 5 min and re-suspended in ~200 μl residual volume. Cells were then infected with NL4-3 at a multiplicity of infection (MOI) of 0.01 and incubated at 37°C in a 5% CO_2_ atmosphere for 4 h. The cells were then washed twice with 10 ml of R20 and 0.5 × 10^6^ cells were plated at a density of 1 M PHA-blast/ml in 48 well plates. Cultures were maintained at 37°C and 5% CO_2_ for 5 days in R20/50 containing different concentrations of BAGN (range: 0, 0.02, 0.2, and 2 mM) and intracellular p24 production was measured by flow cytometry. Briefly, Live/Dead violet fluorescence fixable dead cell stain reactive kit (Invitrogen) was added to cultured cells, followed by extracellular staining with antibodies: CD3-PE-Cy7, CD4-PreCp, and CD8-V500 (BD Biosciences). The cells were fixed and permeabilized (Fix and Perm, Invitrogen) for intracellular staining with the p24 reactive KC57-PE antibody (Coulter). Cells were run on an LSR II instrument (Becton Dickinson) and analysis performed using FlowJo software.

### Production of Viral Stocks in the Presence of BAGN

Two viral stocks of NL4-3 were produced by infecting PHA-blasts, derived from pooled PBMC from three HIV sero-negative donors, in the presence or absence of BAGN. To produce viral stocks grown in the presence of BAGN, 2 days old PHA-blasts were pretreated overnight with 2 mM of BAGN from day 2 to 3. At day 3, 10 million BAGN pretreated PHA-activated cells and 10 million not BAGN pretreated cells were pelleted by centrifugation at 400 *g* for 5 min. Both BAGN pretreated PHA-blast and not pretreated PHA-blast were re-suspended in the remaining media and infected with HIV-1 NL4-3 (MOI 0.004) at 37°C and 5% CO_2_ for 4 h. After the 4-h infection period, cells were washed twice with 10 ml of R20. The NL4-3-infected BAGN pretreated PHA-blast were continuously cultured in R20/50 medium supplemented with 0.2 mM of BAGN at a density of 1 × 10^6^ PBMC/ml in a T25 Falcon flask (Corning). The culture conditions for virus grown in the absence of BAGN were identical, except that no BAGN was added to the cultures. For both conditions, the cultures were followed until p24 levels were >0.5 × 10^6^ pg/ml in the supernatant, using a commercial enzyme-linked immunosorbent assay (ELISA, Fujirebio). To collect viral particles, the supernatants were clarified by centrifugation at 400 *g* for 10 min and stored in aliquots at −80°C until use. Viral stocks were titrated using the TZM-bl cell line and Bright Glo kit (Promega). To this end, the viral stocks were serially fivefold diluted in 96 transparent well plates (NUNC), 10,000 TZM-bl cells were added per well and cultured at 37°C and 5% CO_2_. After 2 days, the Bright Glo reagent was added to each well and luminescence measured on a Fluoroskan Ascent™ Luminometer (ThermoScientific).

### Activation Marker and Coreceptor Expression

Benzyl-2-acetamido-2-deoxy-α-d-galactopyranoside-treated and -untreated PHA-blasts were used to monitor expression of surface markers of cell activation and HIV co-receptor CCR5 and CXCR4. Briefly, PHA-blasts were prepared from 20 HIV-negative donors by stimulation with PHA for 2 days. One-half of the cells from each sample was then treated with 2 mM of BAGN overnight. The next day, cells were stained for viability markers (Live/Dead Fixable Dead Cell Stain kit, Invitrogen) and markers of T cell activation (antibodies: anti-human CD3-APC-H7, CD4-PE-Cy7, CD8-V500, CD25-APC, HLA-DR-FITC, and CD38-PerCP-Cy5.5; BD Biosciences). Cells were fixed and permeabilized (Fix and Perm, Invitrogen) to stain with Ki67-PE (BD Biosciences). Additional aliquots of BAGN-treated and -untreated PHA-blasts were used to stain for viability and T cell subsets as before and for HIV co-receptors (antibodies: anti-human CD184-APC (CXCR4), CD195-PE (CCR5); BD Biosciences). Cells were collected on an LSR II instrument (Becton Dickinson) and analysis was performed using FlowJo software.

### Viral Replication Assay (VRA)

PHA-blast target cells from 10 HIV-uninfected donors, either BAGN-treated or -untreated, and stained for surface activation markers and HIV co-receptors expression, were further used for the VRAs. At day 3, both BAGN-treated and -untreated target cells were infected (MOI = 0.001) with the viral stocks produced with or without BAGN in the culture medium. Cells were cultured for 7 days in R20/50, with or without 0.2 mM BAGN, at 37°C in a 5% CO_2_ containing atmosphere. Secretion of p24 into the culture supernatant was measured by ELISA (Fujirebio) and intracellular p24 production was measured by flow cytometry. Briefly, cells were stained with Live/Dead violet fluorescence fixable dead cell stain reactive kit (Invitrogen) followed by extracellular staining with antibodies: CD3-APC-H7, CD4-PreCp, and CD8-APC (BD Biosciences). The cells were fixed and permeabilized (Fix and Perm, Invitrogen) while staining for HIV Gag p24 using the KC57-FITC antibody (Coulter). As before, cells were collected on an LSR II instrument (Becton Dickinson) and analysis performed using the FlowJo software.

### Virus Outgrowth from PBMC

Pooled PBMC from three HIV sero-negative donors were expanded for 3 days in the presence of PHA and with or without the addition of BAGN on day 2, as described above. On day 3, both BAGN-treated and -untreated PHA-blast, were cocultured with PBMC obtained from HIV-infected, cART-untreated individuals (ratio 1:1). Samples from three elite controllers who maintained undetectable plasma viral load (pVL), four viremic controllers (pVL from 133 to 4,074 copies/ml), and five untreated chronic individuals (pVL from 4,700 to 110,000 copies/ml) were tested. Cocultures were maintained at 37°C in a 5% CO_2_ atmosphere. Every 5 or 7 days, the supernatants were tested for p24 production using a commercial ELISA (Fujirebio) and the cultures fed with fresh, BAGN-treated or -untreated PHA-blasts. Cultures were maintained for maximally 21 days.

### Sequencing of HIV Isolates

The V3 loop region of the HIV *env* gene was sequenced from supernatant of the viral outgrowth cultures using next-generation sequencing. Briefly, viral RNA was extracted using the QIAamp Viral RNA (Qiagen) and the V3 loop region of *env* was retrotranscripted and amplified using the SuperScript^®^ III One-Step RT-PCR System with Platinum^®^ Taq High Fidelity DNA Polymerase (Invitrogen). Primers E80F (CCAATTCCCATACATTATTGTG) and E105R (GCTTTTCCTACTTCCTGCCAC) were used at a concentration of 10 µM in an initial incubation at 52°C for 30 min followed by 30 cycles of: 30 s at 94°C, 30 s at 55°C, and 4 min at 68°C, and a final 4-min incubation at 68°C. The RT-PCR product was amplified in a nested PCR using the Platinum *Taq* DNA Polymerase High Fidelity (Invitrogen), 10 mM of dNTPs, 50 mM MgSO_4_ and primers ES7 (CTGTTAAATGGCAGTCTAGC) and E125 (CAATTTCTGGGTCCCCTCCTGAGG). An initial denaturalization step at 94°C for 2 min was followed by 30 cycles of: 94°C for 30 s, 55°C for 30 s, and 68°C for 4 min, and a final incubation at 68°C for 5 min. The amplicons were then sequenced using the Illumina MiSeq Sequencing platform and a 300 × 2 Paired-End kit. Sequencing was performed by the Genomics Core Facility at *Germans Trias i Pujol* Research Institute. Raw sequences were first filtered with TRIMMOMATIC (29) defining a minimum of 250 bp and a 30:20 quality threshold. Resulting paired-end sequences were merged into single sequences using PEAR software (30) and contaminating human sequences were filtered using bowtie against hg18 human reference. Usearch (31) was used to detect chimeric sequences and to cluster the resulting dataset at a 98% similarity threshold. All unique sequences supported by less than 0.5% of the sequence reads were removed. MUSCLE (32) and FastTree2 (33) were used to build a phylogenetic tree and remove cross-contaminating sequences. MEGA5 (34) was used to construct phylogenetic tree for each of the subject samples, using a neighbor-joining (NJ) approach and bootstrapping (*n* = 100) to obtain support information. Finally, all *env* sequences evaluated for co-receptor tropism using geno2pheno web service, using a FPR threshold of 10% (35).

### HIV Outgrowth from Latently Infected ACH2 Cells

To reactivate HIV from latently infected ACH2 cells, cells were cultured in 24-well plates in RPMI supplemented with 10% FBS and treated with BAGN (0.2 and 2 mM) or DMSO control for 2 h. After treatment, a combination of panobinostat (100 nM) and bryostatin (10 nM) was added to the medium to reactivate latent virus. Cells were cultured for additional 24 h and virus-containing supernatants were clarified by centrifugation, filtered through 45-µm, and stored in aliquots at −80°C. Respective samples were titrated in duplicates in TZM-bl cells as described (36).

## Results

### BAGN Increases the Number of Infected Cells and Virus Production in a Dose-Dependent Manner

**Figure 1:**
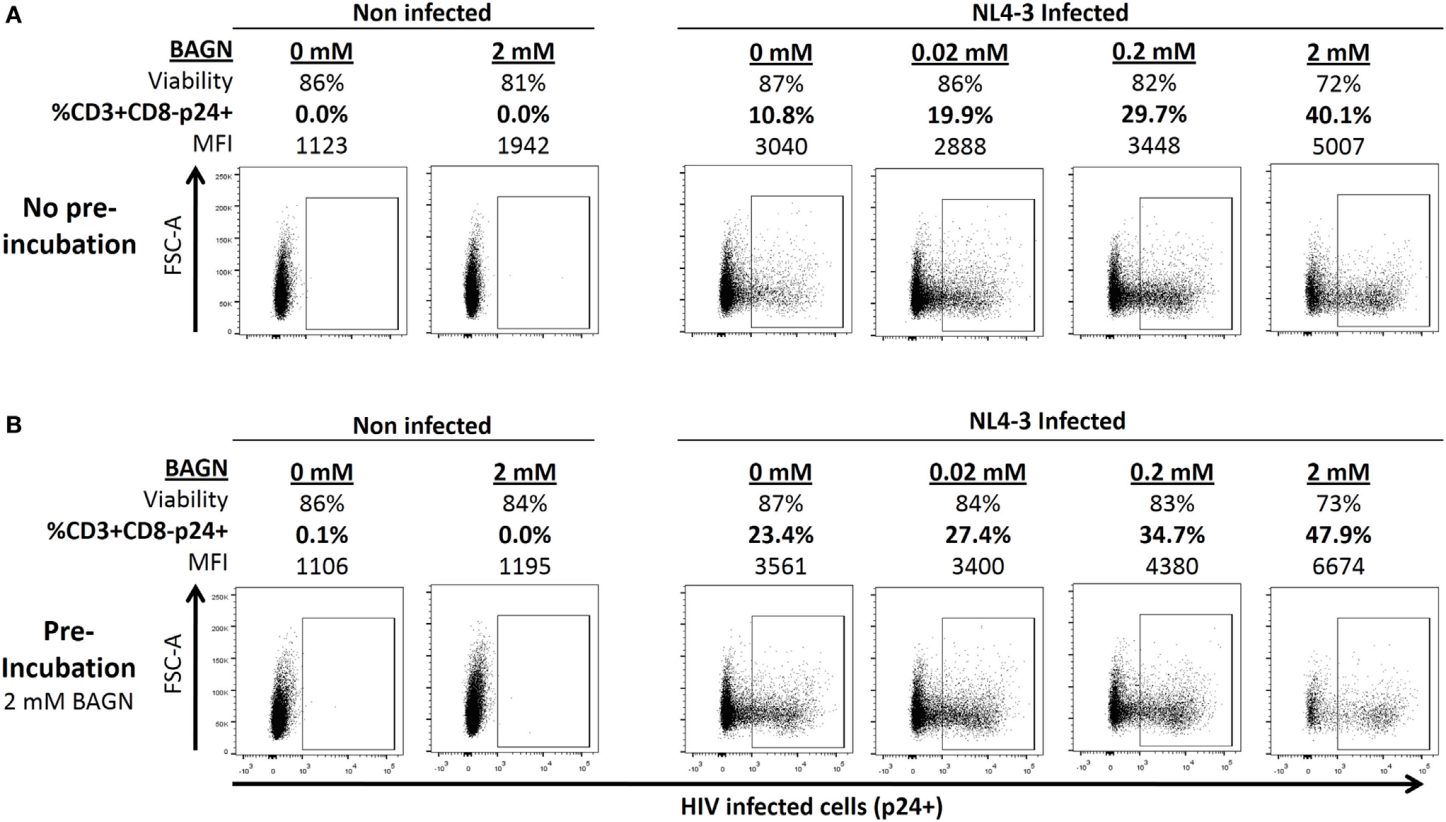
Benzyl-2-acetamido-2-deoxy-α-d-galactopyranoside (BAGN) effect on NL4-3 production is dose dependent and pretreatment of target cells increases infection rates. **(A)** Cells were infected with NL4-3 at a multiplicity of infection of 0.01 and cultured for 5 days. Cell viability, number of p24 positive CD4 T cells, and the median fluorescence intensity (MFI) of p24-specific staining are shown for one representative experiment. **(B)** Same as in panel **(A)** except that target cells were pre-incubated with 2 mM of BAGN overnight before infecting them.

To determine the dependence of HIV replication on O*-*glycosylation, PHA-blasts were infected with HIV-1 NL4-3 in the presence or absence of BAGN. The number of infected cells and the relative amount of intracellular p24 per cell, measured as median fluorescence intensity (MFI), were determined. Different BAGN concentrations, ranging from 0.02 to 2 mM, were added to the culture media after initial infection. Both the percentage and the MFI of infected CD3+ CD8− p24+ cells increased with the concentration of BAGN, reaching the maximum at 2 mM and quadruplicating the number of infected cells (Figure 1A). However, as the viability of target cells decreased at the highest BAGN concentration, a 0.2-mM concentration was used for all subsequent long-term culture conditions.

Besides adding BAGN to the culture of infected cells, we also tested the effect of pretreating target cells with BAGN overnight before exposing them to virus. For this short-term exposure, the higher (2 mM) concentration of BAGN was used, as it did not negatively affect cell viability (Figure 1B). Compared to target cells that were not pretreated with BAGN, an increased number of infected cells was observed (10.8 versus 23.4%), even though the further culture did not contain BAGN. Of note, pretreatment and adding BAGN to the subsequent culture increased the number of infected cells almost fivefold (10.8 versus 47.9%) and showed an increase of the levels of intracellular p24 from a MFI of 3,040 to 6,674.

### BAGN Increases Virus Infectivity and Viral Replication in PHA-Activated T Cells

**Figure 2:**
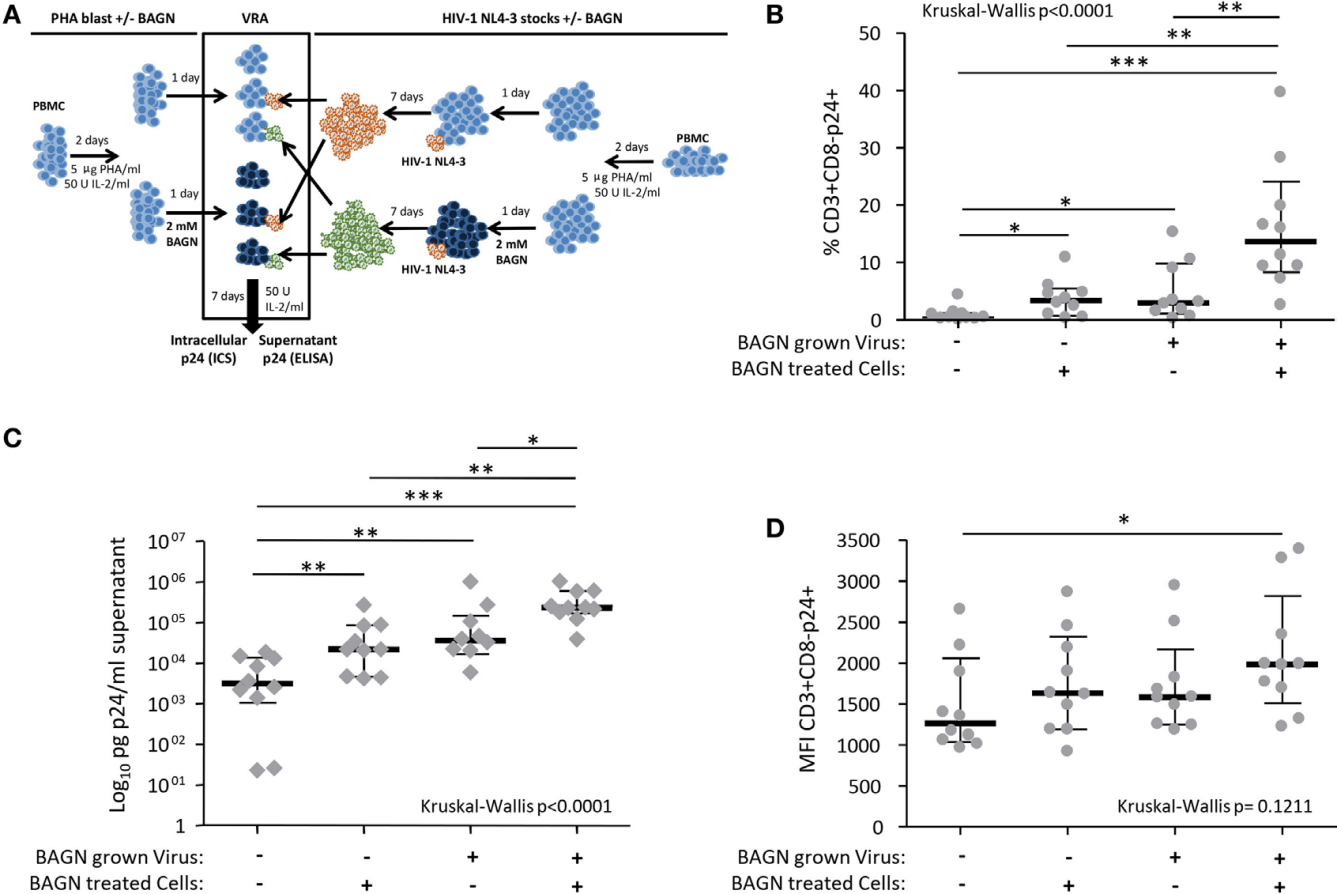
Synergistic effect on human immunodeficiency virus (HIV) production of Benzyl-2-acetamido-2-deoxy-α-d-galactopyranoside (BAGN)-grown virus and culturing target cells with BAGN. **(A)** Experimental outline for generating BAGN-treated/untreated cells (left half), growing virus in the presence/absence of BAGN (right half), and performing the viral replication assay (VRA, central square). **(B)** The number of p24+ CD4 T cells is shown depending on the different culture conditions, median and interquartile ranges are indicated by bars (*N* = 10). *BAGN-grown virus* represents virus stocks previously grown in the presence of BAGN. *BAGN-treated cells* refer to pretreatment and continuous presence of BAGN in the culture. Panels **(C,D)** are same as in panel **(B)** showing the p24 in the supernatant and the median fluorescence intensity (MFI) of infected cells, respectively. Mann–Whitney significance level are indicated by *p < 0.05, **p < 0.001, ***p < 0.0001.

To assess whether BAGN addition to virus cultures could increase viral infectivity and/or replication of HIV, we prepared viral stocks grown in PHA-blasts cultured in the presence or absence of BAGN and used these virus preparations to infect new PHA-blasts, either in the presence or absence of BAGN (Figure 2A). The cultures from these cross-over experiments were tested after 7 days for the percentage of infected CD3+ CD8− cells, their p24 MFI, and the amount of p24 in the culture supernatant. Conducting these experiments in PHA-stimulated PBMC from 10 HIV-uninfected donors showed a consistent and synergetic effect on the percentage of CD3+ CD8- p24+ cells with a median 30-fold increase when using virus stock grown in BAGN-treated cells and adding BAGN to the culture medium compared to entirely BAGN-free conditions (Figure 2B, *p* < 0.0001, Kruskal–Wallis). At the same time, the p24+ MFI of CD3+ CD8− p24+ cells increased with the combination of both treatments (Figure 2D) and led to massively increased levels of p24 in culture supernatants. In the absence of BAGN, the median p24 concentration in the culture supernatant was 3,127 pg/ml, while with the BAGN addition to the culture the median was 22,035 pg/ml, using BAGN-grown virus the median was 36,160 pg/ml and when using both virus grown in BAGN conditions and cell cultures containing BAGN the median reached 232,955 pg/ml. These data show that across a set of 10 HIV-uninfected donors, the combination of using virus grown in BAGN-treated cells, pretreating PHA-activated target cells with BAGN and further culturing cells in the presence of BAGN, can increase median virus levels in the culture supernatant by more than 74-fold.

### BAGN Treatment Modulates Expression of CD25, HLA-DR, and CCR5 on PHA-Activated CD4 T-Cells

**Figure 3:**
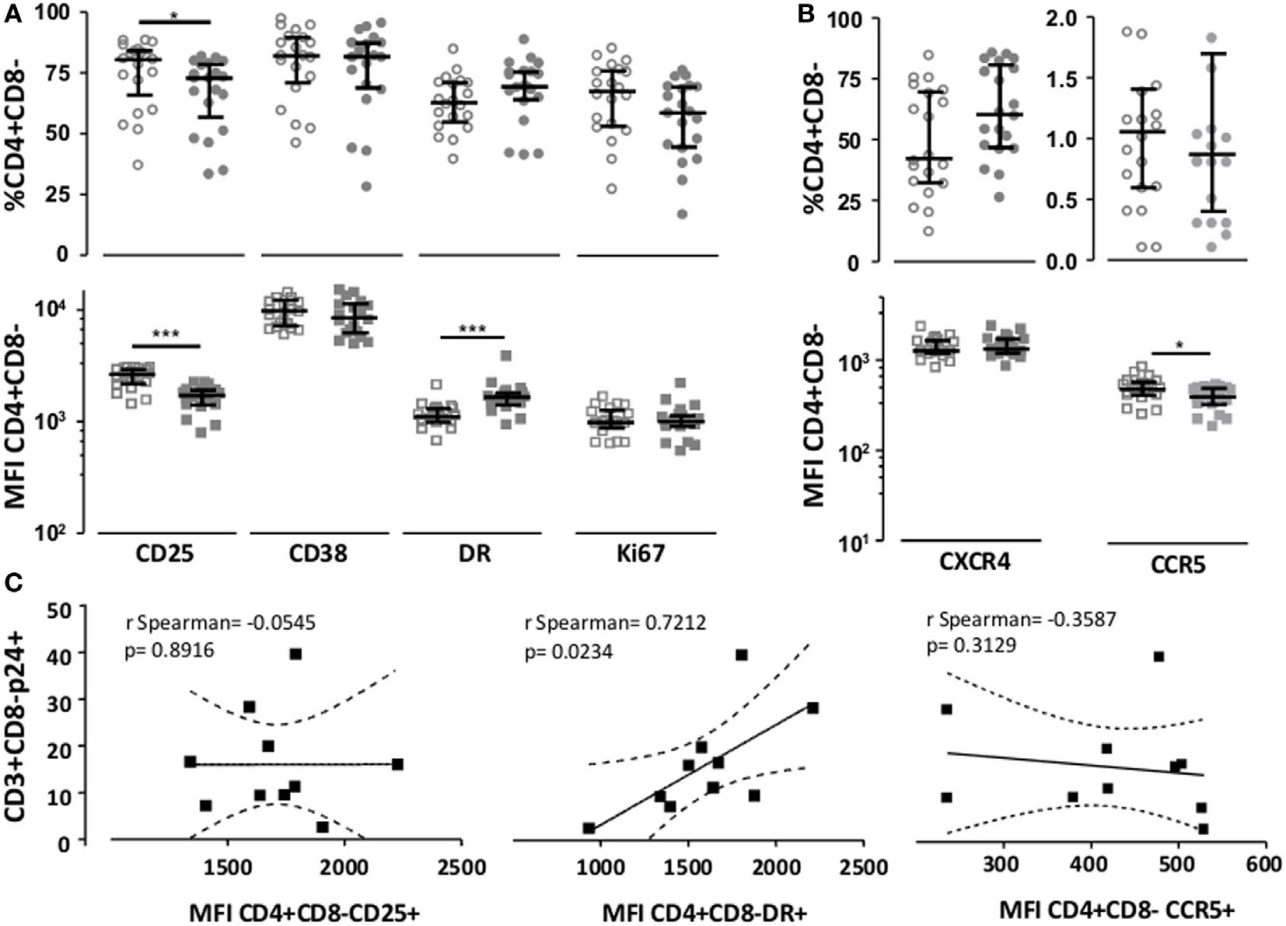
Benzyl-2-acetamido-2-deoxy-α-d-galactopyranoside (BAGN) exposure modulates CD25, HLA-DR, and CCR5 expression. The expression of **(A)** CD4 activation marker and **(B)** human immunodeficiency virus receptors CXCR4 and CCR5 on PHA-activated CD4 T cells was assessed by flow cytometry and expressed as either the percentage of positive cells or median fluorescence intensity (MFI). Open circles or squares indicate cells grown in the absence of BAGN, filled circles or squares show cells grown in the presence of BAGN. Median and interquartile ranges are indicated by bars (*N* = 20). **(C)** Correlation between levels of expression of activation markers and CCR5 co-receptor on BAGN-treated PHA-blasts (MFI) and the number of infected cells (measured as % of CD3+ CD8− p24+ cells). Mann–Whitney significance level are indicated by *p < 0.05, **p < 0.001, ***p < 0.0001.

Since pretreatment of target cells with BAGN increased virus production, even when BAGN was not added to the culture thereafter (Figure 1B), we tested if the differences in infectivity seen could be associated with differences in activation or co-receptor expression attributable to the BAGN treatment right before infection. We compared the expression of activation surface markers on BAGN-pretreated and -untreated cells from 20 HIV-uninfected individuals. The overnight treatment of 2-day old PHA-blasts with 2 mM of BAGN caused a modest decrease in the already high percentage of CD3+ CD4+ CD8− cells expressing CD25 and their median MFI for this marker (Figure 3A). To the contrary, levels of HLA-DR increased by the addition of BAGN (Median MFI of 1,102 without BAGN versus 1,646 with BAGN). The changes in HIV co-receptors, including CXCR4 and CCR5 were measured as well, showing a significant reduction in the level of CCR5 expression (Figure 3B). Ten of the 20 donors assessed for activation and co*-*receptor expression (Figures 3A,B), were included in the infection assays shown in Figure 2, which allowed to compare expression levels of activation markers and HIV co*-*receptors with the percentage of infected CD3+ CD8− T cells (Figure 3C). The data revealed a significant positive correlation between cells staining for HLA-DR and the level of virally infected CD4 T cells (*p* = 0.023, *r* = 0.72, Spearman rank), suggesting that BAGN increased cell activation and thereby allowed for increased virus replication.

### BAGN Treatment Improves *In Vitro* Viral Outgrowth Efficacy

**Figure 4:**
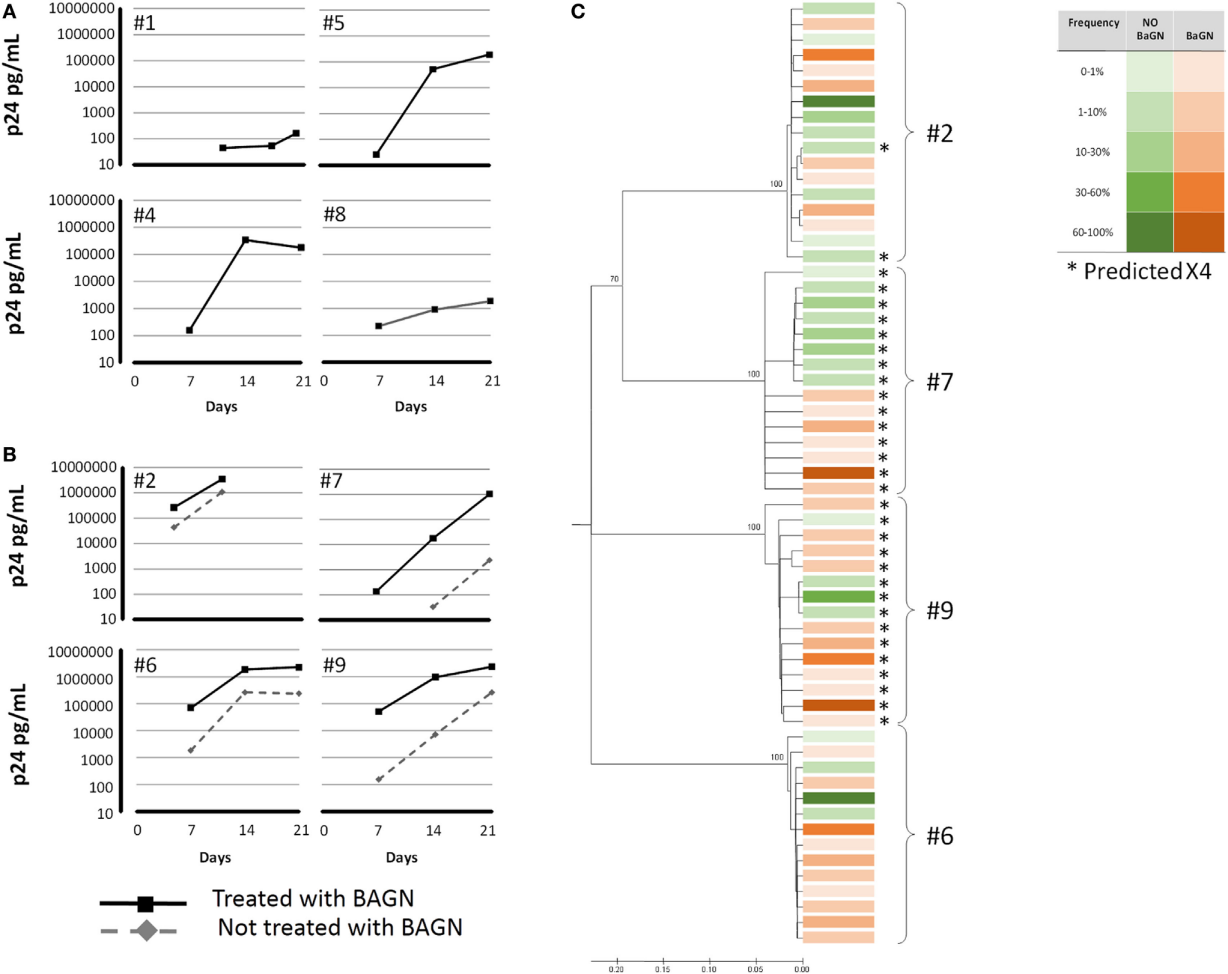
Benzyl-2-acetamido-2-deoxy-α-d-galactopyranoside (BAGN) increases recovery rate in viral outgrowth assays. Peripheral blood mononuclear cells from 12 human immunodeficiency virus-infected individuals were cocultured with PHA-blasts, pretreated and cultured, or not, in BAGN containing medium. Virus recovery failed in four subjects, independent of the culture condition (not shown). In four subjects **(A)**, virus recovery was only possible in the presence of BAGN, and in another four individuals **(B)**, culture with BAGN yielded virus more rapidly and to higher levels compared to cultures without BAGN. **(C)** Virus grown either with or without BAGN conditions was sequenced (envelope V3) and clustered by neighbor-joining tree analysis for the four individuals shown in panel **(B)**. Squares indicate the different sequences, in green those obtained without BAGN in red with BAGN. Intensity of the color indicates relative frequency of the sequence. Sequences predicted to be of X4 tropism are indicated by *.

After seeing the important increase in viral replication after adding BAGN to an *in vitro* infection model using a laboratory strain (NL4-3), we tried to confirm these results in naturally infected PBMC using an HIV outgrowth assay. Various viral outgrowth assays have been developed to measure and characterize viral reservoirs from different tissues (37–41). However, the success rate for growing virus starting with PBMC from HIV-infected individuals varies widely and can be especially problematic in HIV elite and viremic controllers (42). We, thus, tested whether BAGN-mediated inhibition of O*-*glycosylation could also help in improving the rate of virus outgrowth *in vitro*. Overall, 12 PBMC samples were tested obtained from: three elite controllers (undetectable pVL), four viremic controllers (pVL from 133 to 4,074, median 796 copies/ml), three late progressors (pVL from 4,700 to 34,000, median 6,700 copies/ml), one viremic non-progressor (pVL 33,000 copies/ml) and one chronic viremic individual (pVL 110,000 copies/ml). All subjects were off cART at the time of sampling. For the 12 individuals tested, cell cultures were set up either in the presence or absence of BAGN and outgrowth kinetics and viral quasi-species distributions in the outgrowing viral population were compared (Figure 4). Overall, viral outgrowth failed in four of the 12 individuals with and without BAGN addition to the cultures. These included the three elite controllers and one viremic controller with a viral load of 692 copies/ml. In an additional four subjects, including three viremic controllers and one late progressor (pVL 6,700 copies/ml), virus was recovered when BAGN was added but not in BAGN-free conditions. In the remaining four subjects, with viral loads ranging from 4,700 to 110,000 copies/ml, virus was obtained with and without BAGN addition. However, in all samples with detectable virus, higher p24 levels were observed at all time-points in the presence of BAGN compared to cultures without (median p24 concentration at end of culture without BAGN 248,844 pg/ml and with BAGN 2,292,000 pg/ml, Mann–Whitney test *p* = 0.0571). These data show that with the addition of BAGN, viral recovery improved in 66% of tested subjects, including 33% of all individuals where virus was not recovered at all without the addition of BAGN.

To address whether BAGN treatment led to a preferential expansion of specific subpopulations of the individuals’ viral quasi-species, we sequenced the virus recovered from the four individuals that showed growth with and without BAGN addition to the culture. Raw Sequencing data are available at the NCBI/SRA portal with BioProject (PRJNA400540). Based on V3 loop *env* sequences and using a NJ phylogenetic tree, samples from three subjects showed intermingled sequences, indicative of outgrowth of comparable viral populations under the two culture conditions (Figure 4). In one subject (#7), sequences obtained with and without BAGN were clustered separately, although the differences were not supported by high bootstrap values. In addition, V3 loop amino acid sequences were analyzed to predict usage of CXCR4 or CCR5 co-receptor. Except for subject #2 where a minor population was found to possibly be CXCR4-tropic, the viral populations grown with or without BAGN did not differ in their predicted cell tropism.

### BAGN Increases Virus Recovery from Latently Infected Cells Treated with Latency Reactivators

**Figure 5:**
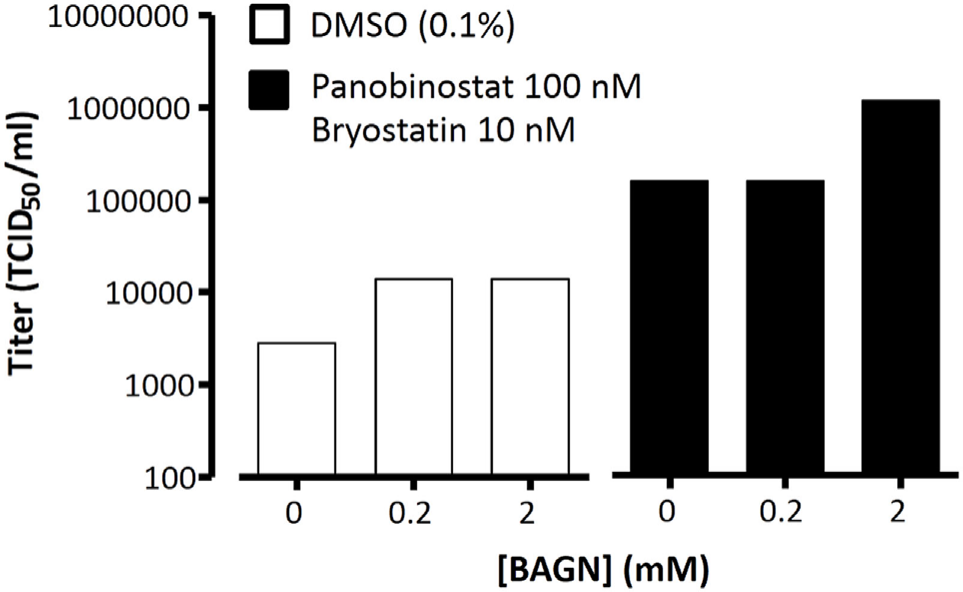
Benzyl-2-acetamido-2-deoxy-α-d-galactopyranoside (BAGN) improves outgrowth of latent virus but only in the presence of latency-reversing agents. ACH2 cells were incubated with DMSO or with panobinostat/bryostatin with increasing doses of BAGN. Virus outgrowth was measured at 24 h by determining the TCID50/ml in duplicates in TZM-bl cells.

Given the superior outgrowth efficacy in expanding virus from total PBMC (Figure 4), we hypothesized that one possible explanation was that BAGN could act as a latency-reversing agent (LRA). To test this hypothesis, we addressed whether BAGN addition could help in expanding reactivated virus in a viral latency model. The ACH2 cell latency model is a system that contains latently integrated HIV DNA with a mutation in the TAR element, which, however, is still responsive to PHA stimulation to reactivate virus (43). Although ACH2 cells constitutively replicate low levels of virus, strong virus growth can be achieved by LRA, such as panobinostat and bryostatin (44–46). When adding BAGN to the combination of panobinostat and bryostatin, a more than four-logs increased titer of infectious virus was obtained within 24 h of culture at the higher concentration of 2 mM BAGN (Figure 5). Although we observed an effect of BAGN alone on viral growth, this effect was limited in the absence of LRA and likely attributable to the low HIV constitutive replication of HIV in ACH2 cells which BAGN magnified (47). These results suggest that BAGN boosts virus production once latent virus has been reactivated by classical LRA, but it does not have a latency-reversing effect on its own.

## Discussion

Glycosylation is the most abundant and diverse posttranslational modification of host proteins and has been studied in the context of viral proteins as well. For HIV, most studies have focused on the role of N*-*linked glycosylation in the folding and antibody recognition of envelope proteins (gp120 and gp41), while little is known about O*-*linked (mucin-like) glycosylation of viral proteins. Even less is known about the possible impact that O*-*glycosylation of host proteins can have on the HIV viral life cycle. We used BAGN, a widely used inhibitor of O-glycosylation in *in vitro* HIV replication cultures to assess its effect on viral growth rates and recovery of residual virus from PBMC.

Benzyl-2-acetamido-2-deoxy-α-d-galactopyranoside treatment increased HIV replication significantly and improved viral outgrowth kinetics in two-thirds of HIV-infected individuals tested, including individuals with low viremia and using a total PBMC culture system without depleting CD8+ T cells (48, 49). For individuals with undetectable viral load, however, the addition of BAGN did not provide a measurable advantage, which does not mean that it could not have positive effects with more refined outgrowth assays or prolonged culture periods (42, 48–50). The increased total virus production in the presence of BAGN appears to be mediated by two factors, an increased number of infected cells in culture and a higher per-cell production of viral particles. The more than 70-fold increase of HIV p24 protein in culture supernatants also indicates that the addition of BAGN did not block HIV budding from infected cells, which could be expected due to the potential inhibition of O*-*glycosylation of host or viral proteins involved in the process. The data also suggest that O*-*glycosylation did not interfere with intracellular transport of viral proteins, as its interruption by BAGN should have reduced the amount of released virus. Of note, the pretreatment of PHA-blast with BAGN additionally increased the percentage of infected cells in the culture, suggesting that BAGN might affect cell susceptibility to infection or that BAGN pretreatment (without further BAGN addition to the culture) has sufficiently long-lasting effects to support more active viral replication once the cell is infected. At the same time, we also observed an increase in the percentage of infected cells in the presence of BAGN or when using virus stocks that were previously grown under BAGN conditions. This suggests that infectiousness of the viral preparation *per se* could have been increased and, once efficient replication has been induced, that the infection can spread through the culture more readily. Aside from higher levels of infectious particles available, this may also be due to an increased susceptibility of cells to HIV infection. The second explanation is probably more plausible since in *in vitro* infection cultures, only a fraction of cells is infected and the susceptibility of cells to infection, rather than the availability of viral particles, may be the limiting factor. Thus, the BAGN treatment may render more cells susceptible to infection spurring overall greater virus production. Alternatively though, virus produced under BAGN conditions may contain viral and/or host proteins in the membrane that, in their de-glycosylated form, provide higher virus infectivity.

The explanation of higher cell susceptibility due to BAGN treatment is also in line with the observation that BAGN had some effects on activation markers expressed on target cells. While CD25 expression was reduced, HLA-DR intensity on BAGN-treated cells was increased and correlated with an increased percentage of infected cells. Although this does not suggest a direct involvement of HLA-DR in virus replication, the findings are supported by previous data showing that HIV profits from cell activation for its replication (51, 52). On the other hand, CCR5 expression was reduced after BAGN treatment, which could lead to a decreased susceptibility to infection with R5 tropism virus. However, this effect was likely not significant as our sequence analyses in the four individuals, from whom we were able to grow virus in the presence and absence of BAGN, showed no shifts in the quasi-species distribution that would support such a notion.

Of interest, two individuals (#6 and #9 in Figure 4) showed a broader distribution of quasi-species when grown under BAGN conditions while the other two individuals showed virus populations of comparable diversity. This raises the possibility that with better outgrowth kinetics, a more representative reflection of the latent viral reservoir could be achieved. This may be critical for personalized cure strategies that depend on a detailed characterization of the viral reservoir populations. More importantly though, BAGN addition allowed us to isolate virus from 1/3 of the tested subjects that did not show any growth in standard assays. As outgrowth efficacies vary between methods used (42, 48, 50, 53), it will be interesting to see how different outgrowth assays can profit equally, or even more, from the addition of BAGN. Furthermore, since virus was grown from multiple individuals under BAGN conditions, the data indicate that BAGN effects are not limited to the laboratory isolate virus used in this study (NL4-3). In this regard, it may be possible to test a wide panel of viruses, including single gene pseudotyped viruses to assess whether sequence variants in viral proteins (for instance envelope) may show differential susceptibility to BAGN-induced effects, be it direct effects on viral proteins or *via* aberrant glycosylation of host (restriction) elements. Such studies may have the potential to identify novel mechanisms of infectivity and viral replication and offer new preventive or therapeutic targets for HIV infection.

From the present data, it is difficult to conclude whether BAGN increases HIV replication by higher virus infectivity, increased cell susceptibility to infection or both. However, Habte et al. have shown that salivary mucins can inhibit HIV-1, which may lead to increased viral replication once these mucins are inhibited by de-O*-*glycosylation (8–12). Similarly, the family of mucin domain TIM proteins have been shown to inhibit HIV-1 release, by causing HIV-1 Gag and mature viral particles to accumulate in the membrane (13). The increased MFI in our flow cytometry-based detection of intracellular p24 initially suggested such an accumulation and potential block of virus release. However, the massively increased p24 levels in the culture supernatant clearly indicate that the virus is being released well from BAGN-treated cells and that the higher MFI may reflect an increased replication and burst size of infected cells. Interestingly, a recent report showed that TIM-1 variants with a 6-amino acid shorter mucin domain were related with delayed HIV progression and a recent study, using our own cohorts, indicates that losing the mucin domain on TIM-1 is associated with relative protection from HIV infection (54, 55). Alternatively, HIV infectivity and cell susceptibility for infection could be increased by more generalizable mechanisms that may affect other viruses as well, including the reduction of the mucin layer associated with membrane glycoproteins, which would increase fusogenicity of the virus or the exposure of the receptors needed for viral infection. The reduction of the mucin layer could also allow the exposure of receptors usually hidden by the mucin layer, like Siglec receptors shorter than Siglec-1, e.g., Siglec-4 and -6 (56–58). This would be in line with recent reports that show founder viruses to carry a reduced (N*-*linked) glycan shield (59–61). Finally, BAGN has also been described to inhibit sialylation in HT-29 cells (22, 28), a modification that can occur on O*-*, N-glycans and glycosphingolipids. Since sialylation occurs in outermost saccharide of formed glycans and since BAGN inhibits the extension of the O-glycan core, sialylation is unlikely to occur in BAGN-treated mucins and impact viral replication. Indeed, alveolar macrophages of HIV-infected individuals have been shown to undergo less O-glycan sialylation (62) and HIV replication is increased after neuraminidase treatment of virus and target cells (63, 64).

Regardless of the detailed mechanisms, our data suggest that glycosylation of structural viral proteins may be a defense mechanisms of the virus (for instance from neutralizing antibodies) that, once inhibited, leads to massively better replication. *In vivo*, however, this defense mechanism may come at a significant cost for efficient viral replication. The data and strategy presented here may help to better understand the balance between these two forces and may help to define their impact on viral replications in untreated individuals. In addition, the data indicate that processes associated with O-glycosylation are critical parameters in the viral life cycle, opening the possibility to identify new therapeutic targets and host proteins whose antiviral function depends on proper glycosylation profiles. More practically though, the data identify modulation of O-glycosylation as a simple strategy to increase replication and recovery of virus *in vitro*. As such, BAGN can help improve viral outgrowth techniques and allow to gain a better knowledge of the viral reservoir populations, a critical component for viral eradication strategies. Since BAGN does not appear to have strong (if any) latency reserving activity, it is also not implied that inhibitors of glycosylation would be a suitable strategy for purging viral reservoirs *in vivo*. Such strategies would possibly also fail since a direct *in vivo* interference with glycosylation could be expected to have serious off-target effects. Still, our data may help to gain an improved picture of the viral reservoir, virus replication kinetics and could thereby critically inform the HIV eradication and cure agenda.

## Ethics Statement

This study was carried out in accordance with the recommendations of the Ethics Committee of the Hospital Universitari Germans Trias i Pujol (Badalona, Spain) with written informed consent from all subjects. All subjects gave written informed consent in accordance with the Declaration of Helsinki. The protocol was approved by the Ethics Committee of the Hospital Universitari Germans Trias i Pujol (Badalona, Spain).

## Author Contributions

AO performed viral replication assays and drafted the first manuscript version. AO and CB performed data analysis and manuscript writing. JM and AM performed HIV outgrowth from latently infected ACH2 cells. AL conducted viral stock titrations. MR performed viral outgrowth experiments. BM and MR-R helped in sample selection and study design. GA and GV assessed O-glycosylation metabolic routes and BAGN inhibitory mechanisms. MC, MN-J and RP performed virus sequencing, phylogenetic analysis, and tropism prediction. Final manuscript version is a collaborative effort of all coauthors.

## Conflict of Interest Statement

The authors declare that the research was conducted in the absence of any commercial or financial relationships that could be construed as a potential conflict of interest.
